# Prognostic impact of admission high-sensitivity C-reactive protein in acute myocardial infarction patients with and without diabetes mellitus

**DOI:** 10.1186/s12933-020-01157-7

**Published:** 2020-10-20

**Authors:** Claudia Lucci, Nicola Cosentino, Stefano Genovese, Jeness Campodonico, Valentina Milazzo, Monica De Metrio, Maurizio Rondinelli, Daniela Riggio, Maria Luisa Biondi, Mara Rubino, Katia Celentano, Alice Bonomi, Nicolò Capra, Fabrizio Veglia, Piergiuseppe Agostoni, Antonio L. Bartorelli, Giancarlo Marenzi

**Affiliations:** 1 Centro Cardiologico Monzino IRCCS, Via Parea 4, Milan, 20138 Italy; 2 Department of Clinical Sciences and Community Health - Cardiovascular Section, University of Milan, Milan, Italy; 3 Department of Biomedical and Clinical Sciences, “Luigi Sacco”, University of Milan, Milan, Italy

**Keywords:** Acute myocardial infarction, Inflammation, High-sensitivity C-reactive protein, Diabetes mellitus

## Abstract

**Background:**

High-sensitivity C-reactive protein (hs-CRP) elevation frequently occurs in acute myocardial infarction (AMI) and is associated with adverse outcomes. Since diabetes mellitus (DM) is characterized by an underlying chronic inflammation, hs-CRP may have a different prognostic power in AMI patients with and without DM.

**Methods:**

We prospectively included 2064 AMI patients; hs-CRP was measured at hospital admission. Patients were grouped according to hs-CRP quartiles and DM status. The primary endpoint was a composite of in-hospital mortality, cardiogenic shock, and acute pulmonary edema. Two-year all-cause mortality was the secondary endpoint.

**Results:**

Twenty-six percent (n = 548) of patients had DM and they had higher hs-CRP levels than non-DM patients (5.32 vs. 3.24 mg/L; P < 0.0001). The primary endpoint incidence in the overall population (7%, 9%, 13%, 22%; P for trend < 0.0001), in DM (14%, 9%, 21%, 27%; P = 0.0001), and non-DM (5%, 8%, 10%, 19%; P < 0.0001) patients increased in parallel with hs-CRP quartiles. The adjusted risk of the primary endpoint increased in parallel with hs-CRP quartiles in DM and non-DM patients but this relationship was less evident in DM patients. In the overall population, the adjusted OR of the primary endpoint associated with an hs-CRP value ≥ 2 mg/L was 2.10 (95% CI 1.46-3.00). For the same risk, hs-CRP was 7 and 2 mg/L in patients with and without DM. A similar behavior was observed for the secondary endpoint when the HR associated with an hs-CRP value ≥ 2 mg/L found in the overall population was 2.25 (95% CI 1.57-3.22). For the same risk, hs-CRP was 8 and 1.5 mg/L in DM and non-DM patients.

**Conclusions:**

This study shows that hs-CRP predicts in-hospital outcome and two-year mortality in AMI patients with and without DM. However, in DM patients, the same risk of developing events as in non-DM patients is associated to higher hs-CRP levels.

## Introduction

Type 2 diabetes mellitus (DM) is a common comorbidity in acute myocardial infarction (AMI), and it is associated with two-fold higher in-hospital and long-term mortality rates and with a higher risk of recurrent cardiovascular events [1–3]. Both DM and atherosclerosis are multifactorial conditions, which share a common inflammatory basis [4]. Indeed, on the one hand, DM is an independent risk factor for AMI and is considered a state of low-grade inflammation [5, 6]. On the other hand, inflammation plays a critical role in all phases of coronary athero-thrombosis, including plaque progression, rupture, and thrombosis leading to AMI [7].

C-reactive protein (CRP), an acute phase protein secreted by the liver, is the most widely used biomarker for detecting inflammatory conditions [8]. The elevation of CRP levels frequently occurs in AMI, and it has been associated with adverse outcomes, including higher risk of major adverse cardiac events, cardiovascular death, chronic kidney disease progression, acute kidney injury, and all-cause mortality [9–13]. To assess cardiovascular risk, physicians have now adopted high-sensitivity CRP (hs-CRP), instead of standard CRP assays that monitor infections and other inflammatory conditions [9]. In particular, in the AMI setting, hs-CRP demonstrated to be a more reliable indicator of outcome than CRP measured through traditional assays [11–15], showing that even a mild increase (≥ 2 mg/L) is of prognostic relevance [16].

Elevated hs-CRP levels in AMI patients may reflect a variable combination of chronic and acute (due to the ongoing cardiac event) inflammation. Since DM is more frequently associated with some degree of chronic inflammation, it is possible that, in AMI patients with DM, hs-CRP has a different prognostic relevance as compared to their non-DM counterpart.

The purpose of this study was to investigate the association between hs-CRP levels, measured at hospital admission, and in-hospital outcome and two-year mortality in a cohort of AMI patients according to DM status.

## Materials and methods

### Study population

This was a prospective, observational study. We enrolled all consecutive patients with AMI (n = 2178), both ST-elevation myocardial infarction (STEMI) and non-ST-elevation myocardial infarction (NSTEMI), admitted to the Intensive Cardiac Care Unit of Centro Cardiologico Monzino in Milan between June 1, 2012 and October 1, 2017. Patients experiencing AMI as a complication of elective percutaneous coronary intervention (PCI) (Type 4a AMI) and those with concomitant systemic inflammatory conditions, including active infections (n = 88) or malignancies (n = 26) were excluded. The study complied with the Declaration of Helsinki, and the Ethics Committee of our center approved the research protocol (n. R520-CCM549). Written informed consent was obtained from all participants.

### Study protocol

Patients were considered as suffering from DM if one of the following conditions were present: personal history of DM reported in clinical record, treatment with glucose lowering drugs, or a glycated hemoglobin value ≥ 6.5% (48 mmol/mol). Glycated hemoglobin was measured at hospital admission in all patients as a part of our routine laboratory package using a method NGSP certified and standardized to the DCCT assay [17].

High-sensitivity-CRP was measured at hospital admission by Cobas^®^ assay (particle-enhanced immunoturbidimetric assay) on Cobas c501 (Roche) [18]. A hs-CRP value ≥ 2 mg/L was considered a sign of inflammation [16].

Study patients received medical treatment and coronary revascularization based on the current standards of care recommended by published guidelines on AMI [19]. Demographical, clinical, biochemical data, and echocardiographic left ventricular ejection fraction (LVEF) were collected at hospital admission. After hospital discharge, all patients were followed-up for 2 years. Patient follow-up was mainly obtained through regularly scheduled outpatient visits or, in a minority of cases, by telephone calls performed by dedicated medical personnel.

The primary endpoint of the study was a composite of in-hospital mortality, cardiogenic shock, and acute pulmonary edema. Cardiogenic shock was defined as persistent systolic arterial pressure  ≤ 80 mmHg and evidence of vital organ hypoperfusion caused by severe left ventricular dysfunction, right ventricular infarction, or mechanical complications of infarction, and not due to hypovolemia, hemorrhage, bradyarrhythmias, or tachyarrhythmias. Acute pulmonary edema was defined as respiratory distress, tachypnea, and orthopnea with rales over the lung fields and arterial oxygen saturation  < 90%. To avoid interference, each patient could only account for one event classification. Two-year all-cause mortality was the secondary endpoint of the study.

### Statistical analysis

A sample size of 2000 patients was calculated under the following assumptions: 10% overall incidence of the primary endpoint [1–3], and an expected odds ratio (OR) increasing by a 1.5 factor from the first to the fourth hs-CRP quartile in the overall population. This sample size allowed an 85% statistical power in assessing a significant difference (α error of 0.05) of the primary endpoint between the two quartiles. Moreover, this sample size allowed a 90% statistical power when an overall incidence of 20% of two-year all-cause mortality was considered [20],with an expected 20% higher mortality risk (hazard ratio [HR] 1.2) between the first and the fourth hs-CRP quartile.

Continuous variables are presented as mean ± SD. Variables with a skewed distribution are presented as median and interquartile ranges. Categorical data are presented as n (%). Trends across hs-CRP quartiles were assessed by ANCOVA and by Mantel–Haenszel Chi square, as appropriate. The association between hs-CRP and study endpoints was assessed by logistic regression analysis. Results are presented as OR with 95% confidence intervals (CI). Cox proportional hazard model was also used to assess HR and 95% CI for two-year mortality associated with hs-CRP quartiles. We calculated the P value for interaction between DM status and hs-CRP quartiles by logistic regression analysis and by Cox proportional hazard model, as appropriate. Pearson coefficient was used to assess the correlation between continuous variables. All analyses were performed in the overall study population as well as in DM and non-DM patients considered separately.

Kaplan–Meier analysis was used to generate time-to-event curves for two-year mortality in patients with hs-CRP < 2 mg/L or ≥ 2 mg/L. Log rank test was used to compare strata.

All analyses were adjusted according to an epidemiological model including the variables most closely associated with prognosis in AMI patients with and without DM [3]: LVEF ≤ 40%, estimated glomerular filtration rate (MDRD equation, based on age, gender, and serum creatinine concentration) ≤ 60 ml/min/1.73 m^2^, and AMI type (STEMI vs. NSTEMI). Moreover, we included in the model previous statin therapy due to its well-known anti-inflammatory effects [21].

Receiver-operating characteristics (ROC) curves were constructed to assess the sensitivity and specificity throughout the concentrations of hs-CRP to predict both primary and secondary endpoints.

Cochran-Mantel–Haenszel estimator was implemented to calculate the adjusted relative risk (RR) of two-year mortality in patients with and without DM.

A bootstrap approach with 2000 resamples was implemented to assess that, in classifying primary and secondary endpoints, the estimated best cutoff of hs-CRP values was consistently higher in DM patients than in non-DM patients.

All tests were 2-tailed, and a P < 0.05 was required for statistical significance. All analyses were performed using SAS version 9.4 (SAS Institute, Cary, NC).

## Results

Two-thousand-sixty-four AMI patients (mean age 67 ± 12 years, 1516 men, 1016 STEMI) were enrolled in the study. Of them, 548 (26%) had DM and 1366 (66%) had hs-CRP levels ≥ 2 mg/L. Inflammation (hs-CRP ≥ 2 mg/L) was more frequent in DM patients than in non-DM patients (74% vs. 64%; P < 0.0001). The baseline clinical characteristics and in-hospital outcomes of patients with and without DM and of those with hs-CRP ≥ and < 2 mg/L are shown in Tables 1 and 2, respectively. Patients with DM were older, more likely to have comorbidities, prior cardiovascular events and higher admission hs-CRP levels than those without DM, despite an almost two-fold higher rate of chronic statin therapy. Moreover, DM patients had a more complicated in-hospital clinical course. Similar differences in clinical characteristics and in-hospital outcomes were observed in patients with hs-CRP ≥ 2 mg/L when compared to those with hs-CRP < 2 mg/L. High-sensitivity-troponin I (hs-TnI) peak value was similar in DM and non-DM patients (43,153 ± 82,894 and 45,392 ± 99,242 ng/L, respectively; P = 0.64) and in patients with and without inflammation (46,427 ± 98,296 and 41,582 ± 89,099 ng/L, respectively; P = 0.28). In the entire population, a significant correlation between admission hs-CRP and hs-TnI peak value was found (r = 0.11; P < 0.0001). This relationship was stronger in non-DM patients (r = 0.12; P < 0.0001) than in DM patients (r = 0.07; P = 0.08).

**Table 1:** Baseline clinical characteristics and in-hospital outcomes of the study patients according to the presence of diabetes mellitus

	DM (n = 548)	Non-DM (n = 1516)	P value
Age (years)	70 ± 11	66 ± 13	< 0.0001
Male sex, n (%)	430 (78%)	1086 (72%)	0.002
Body mass index (kg/m^2^)	28 ± 5	26 ± 4	< 0.0001
Hypertension, n (%)	439 (80%)	897 (59%)	< 0.0001
Smokers, n (%)	166 (30%)	524 (35%)	0.07
Dyslipidemia, n (%)	348 (64%)	682 (45%)	< 0.0001
STEMI, n (%)	237 (43%)	779 (51%)	0.001
Prior MI, n, (%)	204 (37%)	331 (22%)	< 0.0001
Prior CABG, n (%)	110 (20%)	141 (9%)	< 0.0001
Prior PCI, n (%)	199 (36%)	319 (21%)	< 0.0001
LVEF (%)	48 ± 12	51 ± 12	< 0.0001
Time-to-presentation (hours)	12.9 ± 26.5	12.3 ± 26.4	0.64
CA/PCI in hospital, n (%)	495 (90%)	1436 (95%)	0.0005
Laboratory values at hospital admission			
hs-CRP (mg/L)	5.32 (1.86–21.51)	3.24 (1.35–10.03)	< 0.0001
Blood glucose (mg/dl)	202 ± 82	133 ± 42	< 0.0001
HbA1c (%)	7.4 ± 1.7	5.5 ± 0.4	< 0.0001
Serum creatinine (mg/dl)	1.02 (0.8–1.3)	0.92 (0.8–1.1)	< 0.0001
eGFR (ml/min/1.73 m^2^)	73 ± 32	80 ± 25	< 0.0001
Hemoglobin (g/dl)	13 ± 2	14 ± 2	< 0.0001
hs-Tn I (ng/L)	6824 ± 34,686	5875 ± 21,937	0.48
Medication before AMI			
Statins, n (%)	275 (51%)	415 (28%)	< 0.0001
ACEi/ARB, n (%)	271 (49%)	541 (36%)	< 0.0001
Beta-blockers, n (%)	281 (51%)	452(30%)	< 0.0001
Aspirin, n (%)	307 (56%)	443 (29%)	< 0.0001
In-hospital complications			
Death, n (%)	14 (2.6%)	26 (1.7%)	0.22
Cardiogenic shock, n (%)	41 (7%)	76 (5%)	0.03
Acute pulmonary edema, n (%)	92 (17%)	122 (8%)	< 0.0001
Mechanical ventilation, n (%)	28 (5%)	50 (3%)	0.06
Atrial fibrillation, n (%)	79 (14%)	130 (9%)	0.0005
VT/VF, n (%)	28 (5%)	140 (9%)	0.002
High-degree AV block, n (%)	25 (5%)	49 (3%)	0.15
Major bleeding, n (%)	36 (7%)	36 (2%)	< 0.0001
Medication at hospital discharge			
Dual antiplatelet therapy, n (%)	488 (91%)	1444 (97%)	< 0.0001
Statins, n (%)	491 (92%)	1363 (91%)	0.74
Beta-blockers, n (%)	444 (83%)	1138 (77%)	0.002
ACEi/ARB, n (%)	356 (67%)	901 (61%)	0.01

*ACEi* Angiotensin-converting enzyme inhibitors, *ARB* Angiotensin II receptor blockers, *AV* Atrio-ventricular, *CA* Coronary angiography, *CABG* Coronary artery bypass graft, *DM* Diabetes mellitus, *eGFR  *Estimated glomerular filtration rate, *HbA1c* Glycated haemoglobin, *hs-CRP* High-sensitivity C-reactive protein, *hs-TnI* High-sensitivity troponin I, *LVEF* Left ventricular ejection fraction, *MI* Myocardial infarction, *PCI* Percutaneous coronary intervention, *STEMI* ST-segment elevation myocardial infarction, *VT/VF* Ventricular tachycardia/ventricular fibrillation

**Table 2:** Baseline clinical characteristics and in-hospital outcomes of the study patients according to hs-CRP value at hospital admission

	Hs-CRP > 2 mg/L (n = 1366)	Hs-CRP < 2 mg/L (n = 698)	P value
Age (years)	68 ± 12	65 ± 12	< 0.0001
Male sex, n (%)	980 (72%)	536 (77%)	0.01
Body mass index (kg/m^2^)	27 ± 5	26 ± 4	< 0.0001
Hypertension, n (%)	920 (67%)	416 (60%)	0.0004
Diabetes mellitus, n (%)	403 (29%)	145 (21%)	< 0.0001
Smokers, n (%)	474 (35%)	216 (31%)	0.0001
Dyslipidemia, n (%)	680 (50%)	350 (50%)	0.90
STEMI, n (%)	683 (50%)	333 (48%)	0.32
Prior MI, n, (%)	337 (25%)	198 (28%)	0.07
Prior CABG, n (%)	175 (13%)	76 (11%)	0.20
Prior PCI, n (%)	308 (23%)	210 (30%)	0.0002
LVEF (%)	49 ± 12	52 ± 11	< 0.0001
Time-to-presentation (hours)	12.9 ± 25.1	12.2 ± 27.5	0.56
CA/PCI in hospital, n (%)	1260 (92%)	671 (96%)	0.0008
Laboratory values at hospital admission			
hs-CRP (mg/L)	7.37 (3.80–24.11)	1.03 (0.67–1.45)-	
Blood glucose (mg/dl)	157 ± 68	141 ± 52	< 0.0001
HbA1c (%)	6.1 ± 1.3	5.9 ± 1.1	0.001
Serum creatinine (mg/dl)	0.95 (0.8–1.2)	0.92 (0.8–1.1)	0.001
eGFR (ml/min/1.73 m^2^)	76 ± 27	82 ± 26	< 0.0001
Hemoglobin (g/dl)	13 ± 2	14 ± 2	< 0.0001
hs-Tn I (ng/L)	7485 ± 27,414	3468 ± 21,357	0.001
Medication before AMI			
Statins, n (%)	429 (32%)	262 (38%)	0.001
ACEi/ARB, n (%)	528 (39%)	284 (41%)	0.36
Beta-blockers, n (%)	498 (36%)	235 (34%)	0.21
Aspirin, n (%)	496 (36%)	254 (36%)	0.96
In-hospital complications			
Death, n (%)	30 (2.2%)	10 (1.4%)	0.23
Cardiogenic shock, n (%)	90 (7%)	27 (4%)	0.01
Acute pulmonary edema, n (%)	179 (13%)	35 (5%)	< 0.0001
Mechanical ventilation, n (%)	58 (4%)	20 (3%)	0.12
Atrial fibrillation, n (%)	160 (12%)	49 (7%)	< 0.0001
VT/VF, n (%)	101 (7%)	67 (10%)	0.08
High-degree AV block, n (%)	54 (4%)	20 (3%)	0.21
Major bleeding, n (%)	59 (4%)	13 (2%)	0.004
Medication at hospital discharge			
Dual antiplatelet therapy, n (%)	1201 (90%)	623 (91%)	0.64
Statins, n (%)	1214 (91%)	640 (92%)	0.30
Beta-blockers, n (%)	444 (83%)	1138 (77%)	0.002
ACEi/ARB, n (%)	839 (63%)	418 (60%)	0.27

*ACEi* Angiotensin-converting enzyme inhibitors, *ARB* Angiotensin II receptor blockers, *AV* Atrio-ventricular, *CA* Coronary angiography, *CABG* Coronary artery bypass graft, *eGFR* Estimated glomerular filtration rate, *HbA1c* Glycated haemoglobin, *hs-CRP* High-sensitivity C-reactive protein, *hs-TnI* high-sensitivity troponin I, *LVEF* left ventricular ejection fraction, *MI* myocardial infarction, *PCI* percutaneous coronary intervention, *STEMI* ST-segment elevation myocardial infarction, *VT/VF* ventricular tachycardia /ventricular fibrillation

**Fig. 1:**
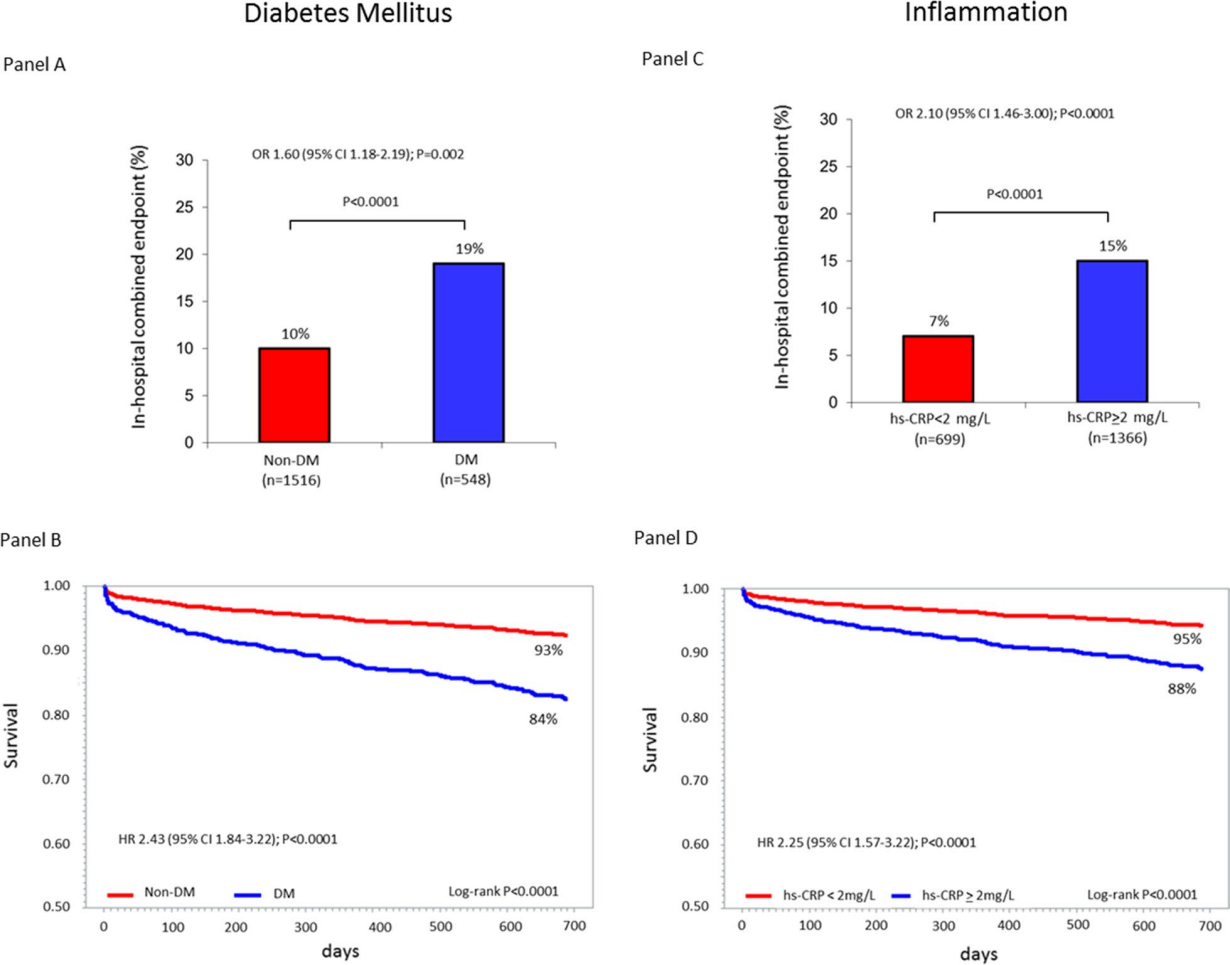
Panel A: incidence of the in-hospital combined clinical endpoint (death, cardiogenic shock, and acute pulmonary edema) in patients with and without diabetes mellitus (DM) and adjusted odds ratio (OR) and 95% confidence interval (CI) associated with DM. Panel B: Kaplan–Meier survival curves stratified by DM status and adjusted hazard ratio (HR) and 95% CI associated with DM. Panel C: incidence of the in-hospital combined clinical endpoint (death, cardiogenic shock, and acute pulmonary edema) in patients with high-sensitivity C-reactive protein (hs-CRP) ≥ and < 2 mg/L and adjusted OR and 95% CI associated with a hs-CRP value ≥ 2 mg/L. Panel D: Kaplan–Meier survival curves stratified by hs-CRP cut-off value (2 mg/L) and adjusted HR and 95% CI associated with a hs-CRP value ≥ 2 mg/L. All analyses were adjusted for left ventricular ejection fraction (≤ or > 40%), estimated glomerular filtration rate (≤ or > 60 ml/min/1.73 m^2^), type of acute myocardial infarction (STEMI vs. NSTEMI) and prior statin use

The incidence of the primary and secondary endpoints in patients with and without DM and in those with hs-CRP ≥ and < 2 mg/L is reported in Fig. 1. Both DM and inflammation had a significantly higher adjusted risk of the two study endpoints.

**Fig. 2:**
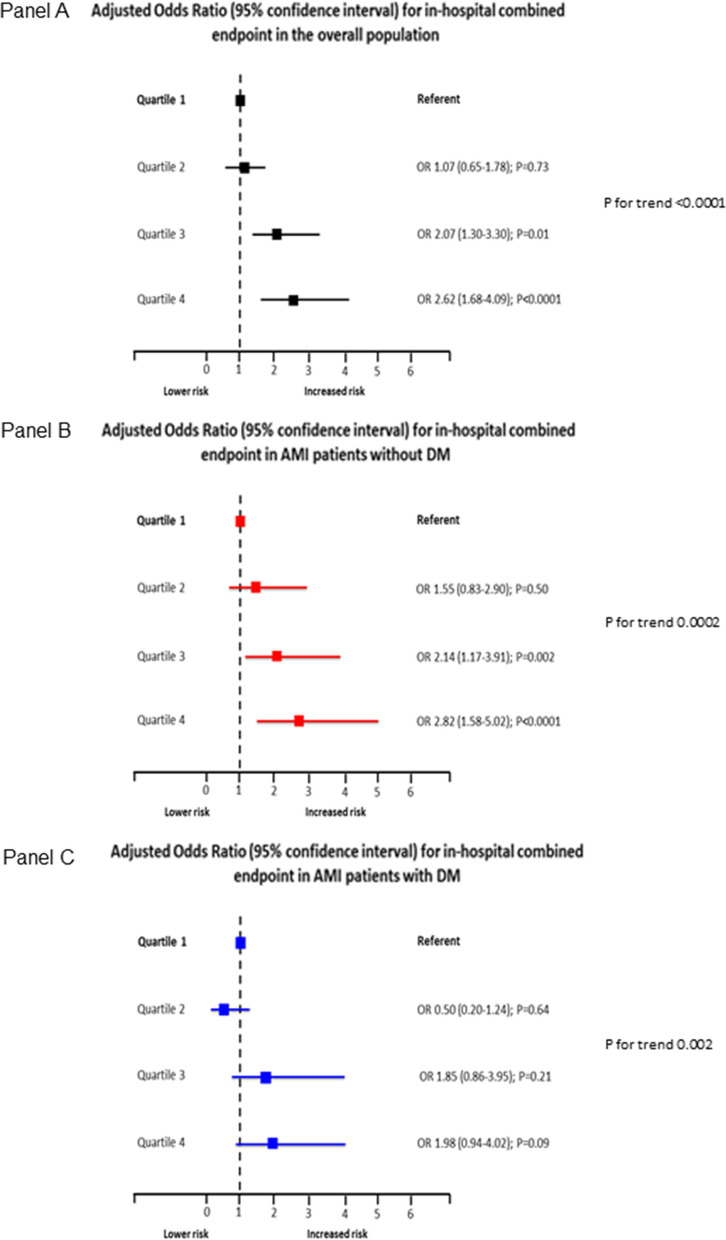
Adjusted odds ratios (OR) and 95% confidence intervals for the primary endpoint according to high-sensitivity C-reactive protein (hs-CRP) level quartiles in the overall study population (Panel A), in patients with diabetes mellitus (DM) (Panel B), and in those without DM (Panel C). Odd ratios and P for trend were adjusted for left ventricular ejection fraction (≤ or > 40%), estimated glomerular filtration rate (≤ or > 60 ml/min/1.73 m^2^), type of acute myocardial infarction (STEMI vs. NSTEMI), and prior statin use. P for interaction between DM status and hs-CRP = 0.36

**Fig. 3:**
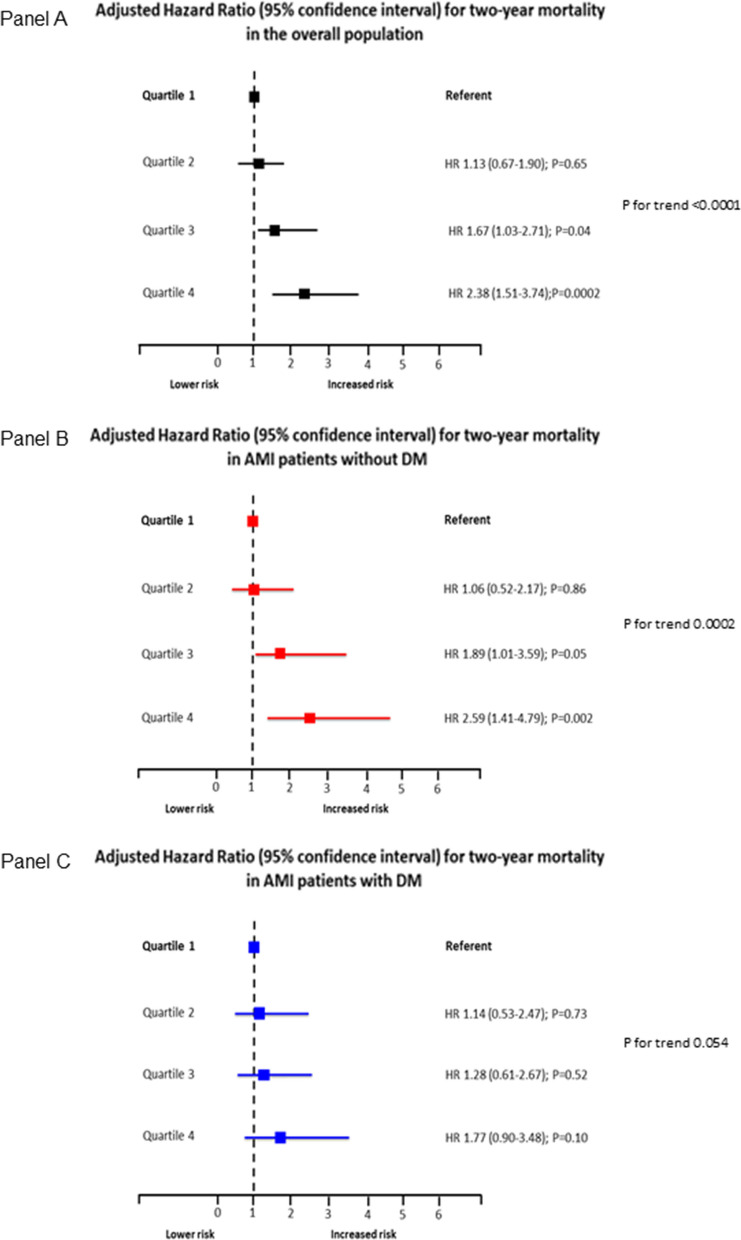
Adjusted hazard ratios (HR) and 95% confidence intervals for the secondary endpoint according to high-sensitivity C-reactive protein (hs-CRP) level quartiles in the overall study population (Panel A), in patients with diabetes mellitus (DM) (Panel B), and in those without DM (Panel C). Hazard ratios and P for trend were adjusted for left ventricular ejection fraction (≤ or > 40%), estimated glomerular filtration rate (≤ or > 60 ml/min/1.73 m^2^), type of acute myocardial infarction (STEMI vs. NSTEMI), and prior statin use. P for interaction between DM status and hs-CRP = 0.02

The incidence of the two study endpoints according to hs-CRP quartiles in the overall population, in DM and non-DM patients is shown in Table 3. In the entire study population, the adjusted risk of the primary endpoint increased in parallel with hs-CRP quartiles (Fig. 2; Panel A). However, this trend was more evident in non-DM patients (Fig. 2; Panel B) than in DM patients (Fig. 2; Panel C). A similar behavior was found when two-year mortality was considered (Fig. 3). In line with this result, a significant interaction between DM status and hs-CRP was found for the secondary endpoint (P = 0.02).

**Table 3:** Primary and secondary endpoint rates according to high-sensitivity C-reactive protein (hs-CRP) quartiles in the overall study population and in patients with and without diabetes mellitus

	Hs-CRP quartiles				
	1 (< 1.45 mg/L)	2 (1.45-3.71 mg/L)	3 (3.72-12.29 mg/L)	4 (> 12.30 mg/L)	P for trend
In-hospital clinical combined endpoint, n (%)					
Overall population	35 (7%)	44 (9%)	68 (13%)	114 (22%)	< 0.0001
Patients with diabetes mellitus	14 (14%)	11 (9%)	29 (21%)	51 (27%)	0.0001
Patients without diabetes mellitus	21 (5%)	33 (8%)	39 (10%)	63 (19%)	< 0.0001
Two-year mortality, n (%)					
Overall population	25 (5%)	36 (7%)	49 (9%)	88 (17%)	< 0.0001
Patients with diabetes mellitus	11 (11%)	18 (15%)	20 (15%)	41 (22%)	0.01
Patients without diabetes mellitus	15 (4%)	18 (5%)	29 (8%)	47 (14%)	<0.0001

The AUCs for hs-CRP in predicting the primary and secondary endpoints in the entire population were 0.66 (95% CI 0.63–0.70) and 0.66 (95% CI 0.62–0.70), respectively. Again, they were higher in non-DM patients (0.66 [95% CI 0.61–0.70] and 0.67 [95% CI 0.61–0.72]) than in DM patients (0.63 [95% CI 0.58–0.68] and 0.61 [95% CI 0.54–0.67]).

**Fig. 4:**
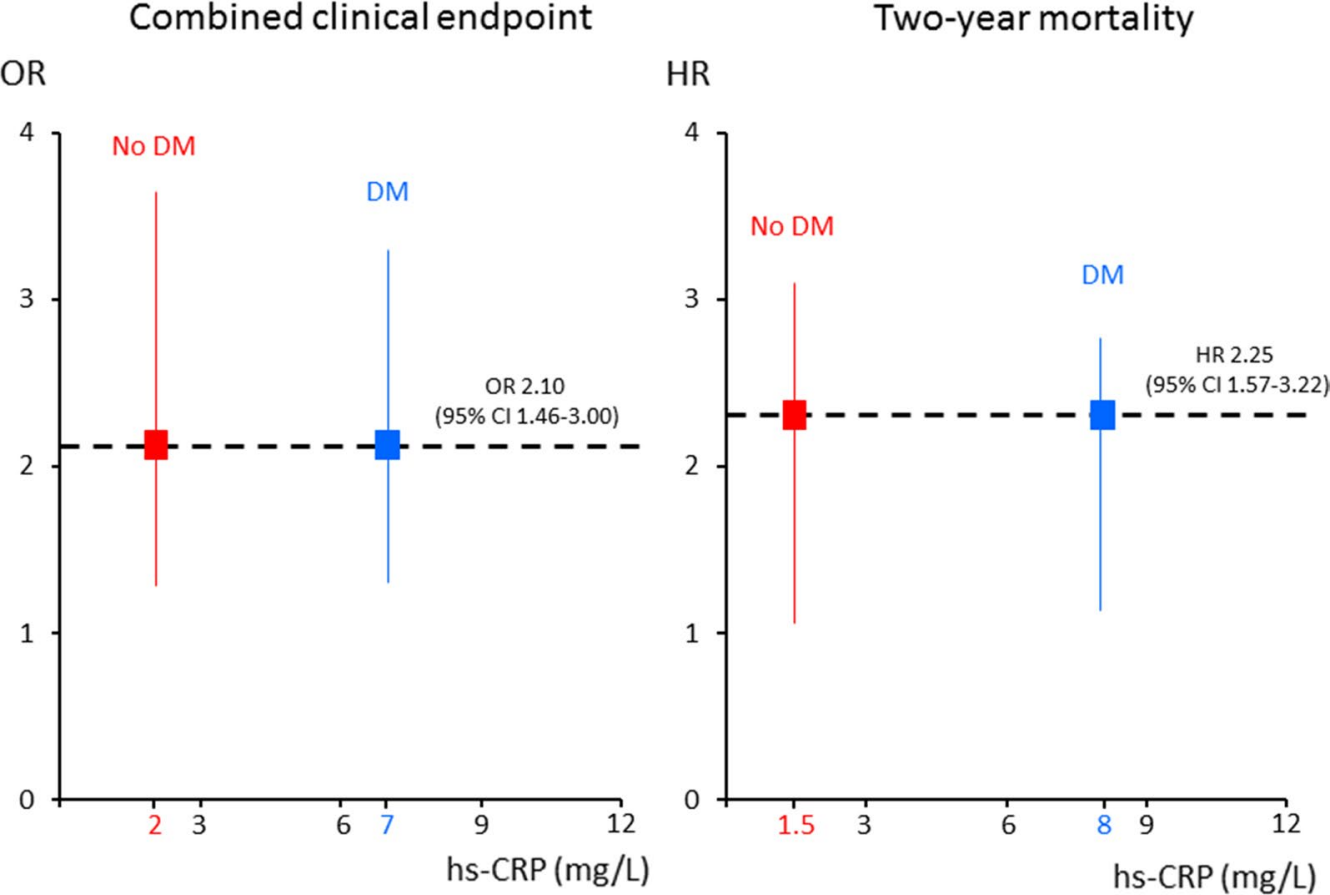
Threshold values of high-sensitivity C-reactive protein (hs-CRP) in patients with and without diabetes mellitus (DM) considered separately, corresponding to the adjusted risk of the primary and secondary endpoints associated with an hs-CRP value ≥ 2 mg/L found in the overall population. *OR* Odds ratio, *HR* Hazard ratio, *CI* Confidence interval

**Fig. 5:**
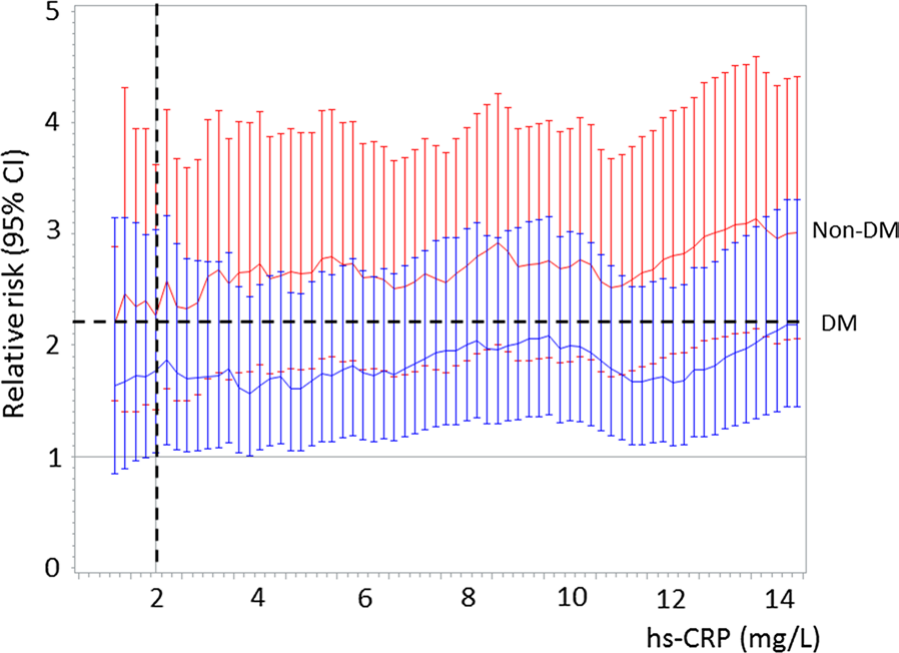
Relative risks and 95% confidence interval (CI) of two-year mortality associated with different high-sensitivity C-reactive protein (hs-CRP) cut-offs in patients with diabetes mellitus (DM) (blue) and in those without DM (red). Relative risk was adjusted for left ventricular ejection fraction (≤ or > 40%), estimated glomerular filtration rate (≤ or > 60 ml/min/1.73 m^2^), type of acute myocardial infarction (STEMI vs. NSTEMI) and and prior statin use. The vertical dotted line refers to hs-CRP value of 2 mg/L. The horizontal dotted line refers to the RR associated with hs-CRP value of 2 mg/L in non-DM patients

The adjusted OR and HR of the primary and secondary endpoint, respectively, associated with an hs-CRP value ≥ 2 mg/L found in the overall population (Fig. 1) corresponded to higher hs-CRP threshold values in patients with DM than in those without DM (Fig. 4). In parallel, at bootstrap analysis, the hs-CRP cutoff values associated with the primary and secondary endpoint risk in DM patients were higher than those of non-DM patients in 74% and 96% of cases, respectively. When computing adjusted RR of two-year mortality in patients with and without DM, its value increased in both groups in parallel with increasing hs-CRP value. However, RR was consistently higher in non-DM patients for any considered hs-CRP level (Fig. 5).

## Discussion

This study supports previous evidence showing that hs-CRP measured at hospital admission in AMI patients is a predictor of in-hospital outcome and long-term mortality. This seems to be true for both DM and non-DM patients. However, we demonstrated that the relationship between the outcomes considered in our study and hs-CRP levels is downshifted in DM patients, who show for each hs-CRP value a lower risk than that of non-DM patients. In other words, in DM patients, the hs-CRP values associated to each event risk were higher than those of non-DM patients.

### Inflammation and DM in AMI

The involvement of inflammation in atherosclerosis and, consequently, in AMI is well established [4–7], as well as the prognostic usefulness of biomarker surrogates, such as hs-CRP, for predicting the risk of mortality and recurrent events [11–16, 22]. Moreover, observational and randomized studies indicated that cardiovascular benefits are more apparent when systemic inflammation is reduced [23, 24]. In particular, the Aggrastat-to-Zocor (A to Z) trial demonstrated that the clinical outcome of patients with acute coronary syndromes significantly improves when the hs-CRP levels are lowered below 2 mg/L [16]. Diabetes mellitus is a multifactorial metabolic disease and growing evidence shows that it is characterized by a state of sub-clinical inflammation [5], as reflected by chronic high levels of hs-CRP [6]. In AMI, patients with DM show a more severe inflammatory condition than those without DM [25], and this may, at least in part, explain their higher short-term and long-term mortality risk [26, 27]. However, whether hs-CRP during AMI carries a different prognostic relevance in DM and non-DM patients is still a controversial issue. Indeed, on the one hand, previous studies showed that CRP is an independent predictor of mortality after AMI in both DM and non-DM patients [28, 29]. On the other hand, Meisinger et al. [28] found no association between CRP and long-term mortality (median 4 years) after AMI in DM patients. However, these studies were retrospective analyses of registries including old study populations (enrolled between 1998 and 2004), they considered patients with an outdated DM definition [28, 29], and, in one study [29], traditional CRP was assessed. More recently, Xia et al. [30] found that CRP predicts three-year mortality in both DM and non-DM patients with AMI. Yet, in this study, the prognostic relevance of CRP was analyzed according to the CRP median value (8.9 mg/L), a cutoff that may encompass patients with the highest degree of inflammation [30]. Thus, the possible different prognostic impact of hs-CRP in AMI patients with and without DM remains unclear.

In our study, we confirmed the presence of a close association between inflammation and DM status in AMI. Indeed, DM patients were more likely to have admission hs-CRP levels ≥ 2 mg/L and had a higher median hs-CRP value than non-DM patients. Moreover, both inflammation and DM status, considered separately, were predictors of in-hospital outcome and two-year mortality, even after adjustment for major confounders. However, when we investigated the relationship between inflammation and outcomes, hs-CRP showed a different behavior in DM and in non-DM patients. In particular, the adjusted risk of the primary and secondary endpoints increased in parallel with hs-CRP quartiles in both groups, but with a more evident trend in non-DM patients. Notably, in the overall population, an hs-CRP value ≥ 2 mg/L was associated with an almost two-fold higher risk of both endpoints. This same risk corresponded to higher hs-CRP values in DM patients, when compared to non-DM patients, thus suggesting that the prognostic relevance of inflammation is maintained also in DM patients but it is shifted towards higher hs-CRP levels. To the best of our knowledge, this is a novel finding, which, if confirmed in future studies, could pave the way for prognostic stratification and intervention strategies tailored according to DM status.

The mechanisms underlying the different prognostic behavior of hs-CRP in DM and non-DM patients are beyond the purpose of the present analysis. However, the following hypothesis can be proposed. In AMI patients, admission hs-CRP level may be considered the result of a variable combination of chronic and acute inflammation. Thus, high hs-CRP levels at hospital admission may not necessarily represent only the inflammatory response associated with AMI severity. Given the well-established association between DM and inflammation, the contribution of chronic inflammation to hs-CRP levels in AMI patients is possibly more relevant in DM than in non-DM patients. Consistently with this theory, a similar hs-TnI peak value, an estimate of myocardial infarct size, was observed in our study in DM and non-DM patients, although the median hs-CRP level was significantly higher in the former group. Moreover, the correlation between hs-CRP levels and hs-TnI peak value was closer in non-DM than in DM patients.

Another intriguing issue is represented by the mechanisms underlying the association between hs-CRP and in-hospital outcome in AMI. In this regard, there is growing evidence that inflammation in AMI is not only a marker of AMI severity but it may directly exacerbate the cardiac dysfunction [31–33]. Indeed, in conditions characterized by acute systemic inflammation—such as severe burn, trauma, or sepsis—cardiac cell death is rare but reversible cardiac myocyte injury often occurs resulting in a transient depression of myocardial contractility [31–33]. Notably, the most important mediators of the inflammatory process, like tumor necrosis factor-α, interleukin-1β, and interleukin-6, have been shown to have a negative inotropic effect on cardiac contractility [31, 32]. Moreover, an association between elevation of inflammatory markers and myocardial reperfusion injury has been reported in AMI [34]. On this account, we considered a combined in-hospital clinical endpoint including acute pulmonary edema, cardiogenic shock, and death, which are clinical equivalents of acute ventricular dysfunction.

### Study clinical implications

Our study may have some potential clinical implications. Firstly, in AMI patients, hs-CRP allows physicians to identify high-risk patients. This is true also for DM patients, in whom, however, a higher hs-CRP threshold than that usually considered (2 mg/L) should be identified to improve risk stratification. This concept is further suggested by the fact that, in our study population, a significant interaction was found between DM status and hs-CRP when long-term mortality was considered. Moreover, the RR of two-year mortality was constantly lower in patients with DM than in those without DM at each given hs-CRP level. For instance, the two-year mortality RR of a non-DM patient with hs-CRP level of 2 mg/L was similar to that of a DM patient with hs-CRP level of 14 mg/L (Fig. 5). Secondly, as hs-CRP has been recently considered a potential therapeutic target in AMI, DM status should be taken into account when anti-inflammatory therapeutic strategies are investigated. The Canakinumab Antiinflammatory Thrombosis Outcome Study (CANTOS) trial showed that, among patients with prior AMI and hs-CRP ≥ 2 mg/L, treatment with a monoclonal antibody targeting interleukin-1β is associated with fewer cardiovascular events [23]. However, in the CANTOS trial, the beneficial effects, in terms of cardiovascular endpoints, were mainly observed in non-DM patients, with a non-significant risk reduction in those with DM [24]. This highlights the possible need of a different hs-CRP cutoff value for the identification of high-risk AMI patients with DM who may benefit the most from an anti-inflammatory therapeutic strategy. Novel therapeutic approaches aiming at reducing hs-CRP levels during AMI are also under investigation, and preliminary experimental and clinical data are being reported on the use of apheresis in this clinical setting [35, 36]. This strategy demonstrated to rapidly and safely lower hs-CRP levels by about 50%, independently of the initial concentration [35]. Interestingly, this reduction was associated with a smaller infarct size in animal models [36].

### Study strengths and limitations

The strengths of our study include its prospective design, a well-characterized population, and a special focus on the relationship between inflammation and DM status in AMI. However, some limitations warrant mention. Firstly, we evaluated an AMI population admitted to a single center and treated, in most cases, with PCI. As this therapeutic strategy may have influenced the results of our study, the overall applicability of our findings to AMI patients not undergoing coronary revascularization needs to be clarified. Moreover, the promptness, extent, and efficacy of myocardial revascularization was not assessed as a confounder event. Secondly, because of the observational nature of the study, a cause-effect relationship between hs-CRP and outcomes cannot be established. Moreover, we did not evaluate the effectiveness of and the adherence to pharmacological treatment, in particular of lipid- and glucose-lowering therapies, during follow-up. Indeed, previous studies reported that the prognostic effect of admission hs-CRP is attenuated in high-intensity statin users [37] and in patients with optimized glycometabolic control [38]. Thirdly, the association between hs-CRP levels at admission and the duration and treatment of DM was not investigated, and it should be considered as a possible bias. Fourthly, we measured only hs-CRP; however, it is possible that other inflammation indexes, such as the CRP to albumin ratio, may be more accurate in predicting outcomes in AMI patients [39, 40]. Fifthly, we measured hs-CRP levels only at admission. As the inflammatory response in AMI begins within hours and peaks after several days [41], hs-CRP levels at other time points might better reflect the magnitude of the acute inflammatory process. Finally, several potential confounding factors associated with chronic inflammation, such as thickened epicardial adipose tissue [42] and fibrinogen concentration [43], were not evaluated in our study.

## Conclusions

The results of this study show that hs-CRP level measured at hospital admission predicts in-hospital outcome and two-year mortality in AMI patients with and without DM. However, in patients with DM, the same risk of developing events as in non-DM patients is associated to higher hs-CRP levels.

## Data Availability

The datasets generated and/or analyzed during the current study are not publicly available, as per internal protocol, but are available from the corresponding author on reasonable request.

## Abbrevations

AMI: Acute myocardial infarction
CAD: Coronary artery disease
CI: Confidence intervals
CRP: C-reactive protein
DM: Diabetes mellitus
OR: Odds ratio
hs-CRP: High-sensitivity-C-reactive protein
HR: Hazard ratio
LVEF: Left ventricular ejection fraction
NSTEMI: non-ST-elevation myocardial infarction
PCI: Percutaneous coronary intervention
ROC: Receiver-operating characteristics
RR: Relative risk
STEMI: ST-elevation myocardial infarction
